# Modeling Congenital Hyperinsulinism with *ABCC8*-Deficient Human Embryonic Stem Cells Generated by CRISPR/Cas9

**DOI:** 10.1038/s41598-017-03349-w

**Published:** 2017-06-09

**Authors:** Dongsheng Guo, Haikun Liu, Aynisahan Ruzi, Ge Gao, Abbas Nasir, Yanli Liu, Fan Yang, Feima Wu, Guosheng Xu, Yin-xiong Li

**Affiliations:** 1 Institute of Public Health, Guangzhou Institutes of Biomedicine and Health, Chinese Academy of Sciences, Guangzhou, China; 2 University of Chinese Academy of Sciences, Beijing, China; 3 Key Laboratory of Regenerative Biology, South China Institute for Stem Cell Biology and Regenerative Medicine, Guangzhou Institutes of Biomedicine and Health, Chinese Academy of Sciences, Guangzhou, China; 4 Guangdong Provincial Key Laboratory of Biocomputing, Guangzhou Institutes of Biomedicine and Health, Chinese Academy of Sciences, Guangzhou, China

## Abstract

Congenital hyperinsulinism (CHI) is a rare genetic disorder characterized by excess insulin secretion, which results in hypoglycemia. Mutation of sulfonylurea receptor 1 (SUR1), encoded by the *ABCC8* gene, is the main cause of CHI. Here, we captured the phenotype of excess insulin secretion through pancreatic differentiation of *ABCC8*-deficient stem cells generated by the CRISPR/Cas9 system. *ABCC8-*deficient insulin-producing cells secreted higher insulin than their wild-type counterparts, and the excess insulin secretion was rescued by nifedipine, octreotide and nicorandil. Further, we tested the role of SUR1 in response to different potassium levels and found that dysfunction of SUR1 decreased the insulin secretion rate in low and high potassium environments. Hence, pancreatic differentiation of *ABCC8-*deficient cells recapitulated the CHI disease phenotype *in vitro*, which represents an attractive model to further elucidate the function of SUR1 and to develop and screen for novel therapeutic drugs.

## Introduction

Congenital hyperinsulinism (CHI) refers to a group of rare genetic disorders that are characterized by excess insulin secretion by pancreatic β-cells. As insulin is a key hormone in the regulation of blood glucose levels, excess insulin secretion leads to severe and persistent hypoglycemia^1^. Persistent hypoglycemia can induce jitteriness, lethargy, unresponsiveness and seizures and can increase the risk of brain injury^2^. The prevalence of CHI has increased from 1:50000 births to 1:2500 in the general population^3^. Therefore, a more relevant and specific disease model system that can recapitulate human CHI pathogenesis is desired for studying the disease mechanism and developing effective therapies.

The molecular mechanisms of CHI involve anomalies in key genes that regulate insulin secretion from β-cells, including *ABCC8, KCNJ11, GCK, SCHAD, GLUD1, SLC16A1, HNF1A, HNF4A*, and *UCP2* ^4^. The most common cause of CHI is inactive mutations of the ATP-sensitive potassium (K_ATP_) channel genes *ABCC8* and *KCNJ11*, which encode the sulfonylurea receptor 1 (SUR1) protein and inwardly rectify potassium channel (Kir6.2) proteins, respectively. Such K_ATP_ mutations are responsible for approximately 36.3% of all CHI cases^5^. To date, more than 150 *ABCC8* mutations and 24 *KCNJ11* mutations have been reported^6, 7^, which can cause continuous depolarization^8^ and Sar1-GTPase-dependent ER exit^9^ resulting in excess insulin secretion.

Although multiple genetic mouse models of CHI have been developed^10–12^, stem cells-based CHI models are still lacking. The CRISPR/Cas9 system has recently emerged as a powerful and highly efficient genome engineering tool^13^. The combination of the CRISPR/Cas9 system and human pluripotent stem cells provides new approaches for generating *in vitro* human disease models^14–16^, allowing for the opportunity to study rare human genetic diseases and screen for possible therapeutic drugs.

In this report, we modeled the phenotype of excess insulin secretion of CHI with *ABCC8*-deficient ES cell and pancreatic beta cell differentiation. The insulin-producing cells that differentiated from the *ABCC8*-deficient stem cells exhibited higher insulin secretion. Excess insulin secretion was rescued by drugs used for CHI treatment. We also tested the response of *ABCC8*-deficient cells to different potassium media and found that the *ABCC8* mutation decreased the insulin secretion rate.

## Results

### *ABCC8-*deficient insulin-producing cells demonstrated higher insulin secretion

We previously reported the generation of CRISPR/Cas9 system-mediated mutated *ABCC8* heterozygous (A2, *ABCC8* ^+/−^, *ABCC8* ^+/+1^, 1 bp insertion at 167 locus of CDS) and homozygous (A4, *ABCC8* ^−/−^, *ABCC8* ^Δ*22*/Δ*22*^, c.167-188 del) cell lines from human ES cells^17, 18^ in which the *ABCC8* mutation did not affect pluripotency or differentiation potential *in vitro*, and the cells contained a normal karyotype. Furthermore, we did not detect any off-target at 8 potential off-target sites, indicating that these isogenic cells could provide an ideal cell model for CHI research.

**Figure 1:**
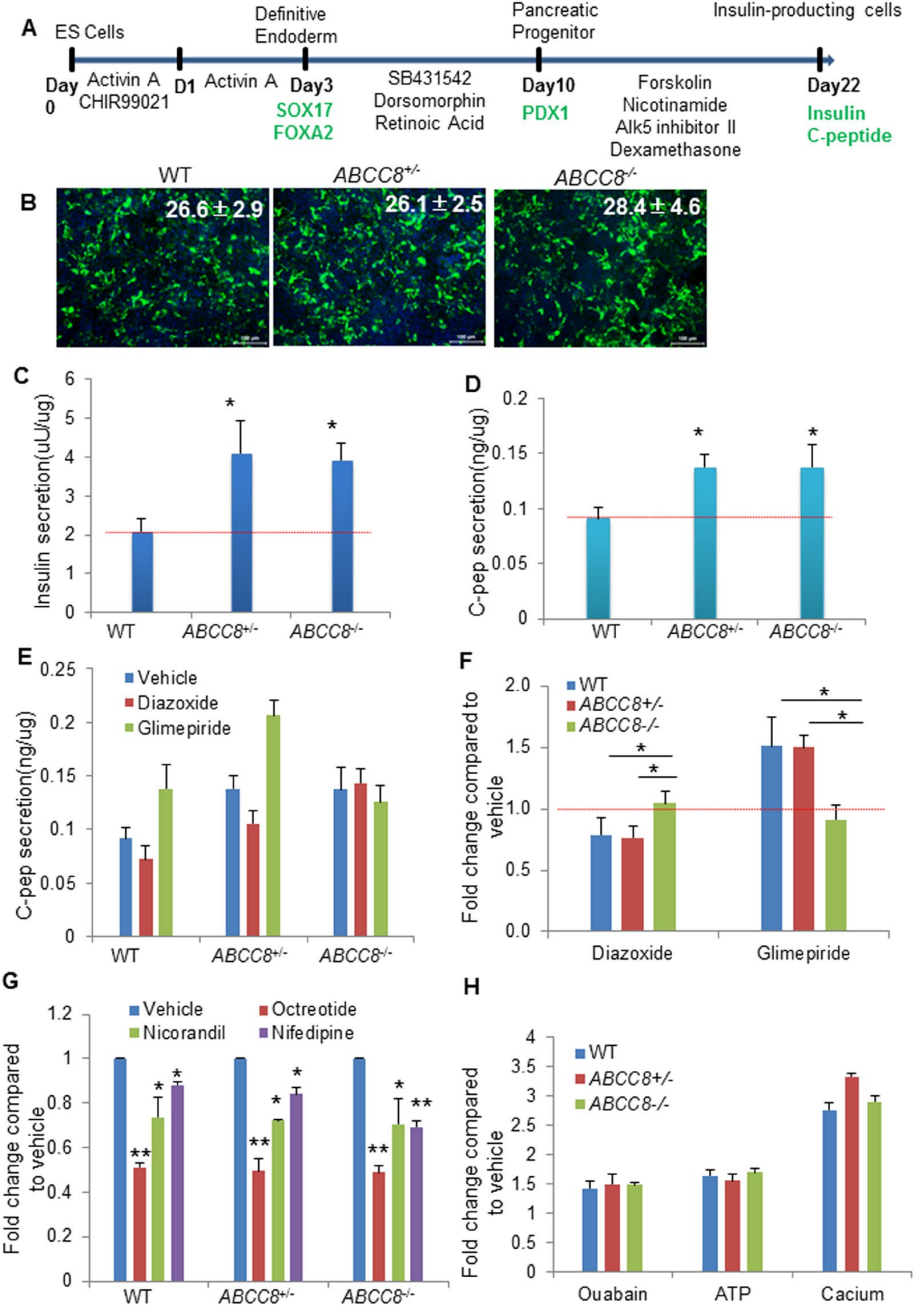
*ABCC8*-deficient insulin-producing cells exhibited greater insulin secretion and insulin secretion responses to the various stimulations. (**A**) Schematic of directed differentiation from pluripotent stem cells to insulin-producing cells via the small molecule-based method. (**B**) Immunofluorescence for insulin at the final stage of differentiation. *ABCC8*-deficient cells demonstrated similar differentiation efficiency as wild-type cells. (**C**) ELISA analysis for insulin content in the supernatant. *ABCC8*-deficient cells demonstrated greater insulin secretion than wild-type cells. (**D**) ELISA analysis for C-peptide content in the supernatant. *ABCC8*-deficient cells demonstrated greater C-peptide secretion than wild-type cells. (**E**) ELISA analysis for C-peptide levels in the presence of vehicle, diazoxide and glimepiride. (**F**) The fold change of C-peptide content after diazoxide and glimepiride stimulation. Diazoxide and glimepiride decreased and increased C-peptide secretion in wild-type and heterozygous mutated cells, respectively. Neither diazoxide nor glimepiride had an effect on homozygous mutated cells. (**G**) The fold change of C-peptide content after octreotide, nicorandil and nifedipine treatment. Octreotide, nicorandil and nifedipine decreased insulin secretion in wild-type, heterozygous mutated and homozygous mutated cells. (**H**) The fold change of C-peptide content after ouabain, extracellular ATP and calcium chloride treatment.

The major clinical symptom of CHI is excess insulin secretion in the blood. To model the phenotype of excess insulin secretion *in vitro*, we differentiated pancreatic beta cells from wild-type and mutated heterozygous or homozygous *ABCC8* cell lines. For specific differentiation towards pancreatic beta cells, we followed a previous protocol with slight modifications^19^ to simulate normal pancreatic development through three main phases: definitive endoderm (DE), pancreatic progenitors (PPs) and insulin-producing cells (IPCs) (Fig. 1A). The expression of markers corresponding to the three phases, FOXA2 and SOX17 for DE, PDX1 for PPs, insulin and C-peptide for IPCs, was verified by immunofluorescence (Supplementary Fig. 1A,B,C). The insulin-producing cells at the end of the final differentiation stage were measured by immunofluorescence and flow cytometry (Fig. 1B; Supplementary Fig. 1D). *ABCC8*-deficient cells and normal cells shared similar differentiation efficiencies in that approximately 25% of terminal phase cells were insulin-positive, indicating that *ABCC8* deficiency does not affect differentiation toward insulin-producing cells. Next, we tested the amount of insulin secreted by the cells in the supernatant in Krebs-Ringer bicarbonate HEPES (KRBH) buffer. The normal insulin content per unit protein for wild-type cells was 2.09 μU, while higher levels of insulin were measured for the *ABCC8* mutants corresponding to 4.09 μU for *ABCC8* ^+/−^ and 3.91 μU for *ABCC8* ^−/−^ cells (Fig. 1C). We also measured C-peptide content in the supernatant, which exhibited the same trend as insulin content: C-peptide content per unit protein for both of the *ABCC8* ^+/−^ and *ABCC8* ^−/−^ cells reached 0.14 ng, which was significantly increased compared with 0.09 ng for wild-type cells (Fig. 1D). These results demonstrated that *ABCC8*-deficient cells secreted insulin and C-peptide at higher levels than wild-type cells, indicating that we created a phenotype of excess insulin secretion *in vitro*. Next, we measured the release of human C-peptide, a by-product of endogenous insulin biosynthesis that is secreted in an equimolar ratio to insulin^20^, as an indicator of insulin secretion.

To test whether SUR1 protein loses its function in *ABCC8*-deficient cells, we tested the effects of the two most widely used modulators, diazoxide and glimepiride, on insulin secretion. Diazoxide is a benzothiazine derivative that acts on the SUR1 subunit^21^ to activate K_ATP_ channels. Diazoxide is an agonist of K_ATP_ channels that is widely used for decreasing insulin secretion in CHI patients^22^. Glimepiride is a sulfonylurea drug that blocks the SUR1 subunit of K_ATP_ channels^23^ and, in turn, is used to increase insulin secretion in diabetic patients^24, 25^. Both diazoxide and glimepiride function in the presence of SUR1 protein. Our results demonstrated decreased insulin secretion in wild-type and *ABCC8* ^+/−^ cells after application of diazoxide but no effect on *ABCC8* ^−/−^ cells. The fold changes observed in wild-type, *ABCC8* ^+/−^ and *ABCC8* ^−/−^ cells were 0.79, 0.76 and 1.04, respectively. In contrast, glimepiride increased insulin secretion in wild-type and *ABCC8* ^+/−^ cells but had no effect on *ABCC8* ^−/−^ cells. The fold changes observed in wild-type, *ABCC8* ^+/−^ and *ABCC8* ^−/−^ cells were 1.51, 1.50 and 0.94, respectively (Fig. 1E,F). These results suggest a loss of function of SUR1 protein in CRISPR/Cas9-mediated *ABCC8* mutants. *ABCC8* ^+/−^ cells may provide a suitable *in vitro* model for screening drugs that can be used to treat CHI patients who are unresponsive to diazoxide.

### Excess insulin secretion by *ABCC8-*deficient ES-IPCs can be rescued by nicorandil, nifedipine and octreotide

The process of insulin secretion is dependent on K_ATP_ channels. High-energy ATP molecules that are generated during carbohydrate metabolism increase the intracellular ATP:ADP ratio, leading to the closure of KATP channels and then causing depolarization of the cell surface membrane. Voltage-gated calcium ion channels open in response to depolarization, and calcium ions move into the cell, inducing insulin secretion^26^. Somatostatin has been proven to inhibit insulin secretion^27^. Nifedipine, a calcium channel antagonist, and octreotide, a somatostatin analogue, have both been used to treat CHI patients^22, 28–30^. Nicorandil, a potassium channel activator^31^, activates K_ATP_ channels^32^. Treatment of wild-type cells with octreotide, nicorandil and nifedipine induced fold changes of 0.51, 0.74 and 0.88 in C-peptide secretion, indicating their effect on decreasing insulin secretion. Therefore, it is interesting to determine whether these drugs can rescue excess insulin secretion in *ABCC8-*deficient cells. We found that octreotide, nicorandil and nifedipine decreased insulin secretion in *ABCC8*-deficient cells, inducing fold changes of 0.50, 0.72, and 0.84 for heterozygous mutated cells and 0.49, 0.71, and 0.69 for homozygous mutated cells, respectively (Fig. 1G). Our results showed that nicorandil, nifedipine and octreotide, which function at different insulin secretion stages, can decrease insulin secretion in *ABCC8*-deficient cells. These data demonstrated that our *ABCC8* mutants provide an ideal model of CHI and could be used for drug screening.

### No change in extracellular ATP-, calcium- and ouabain induced insulin secretion in *ABCC8-*deficient cells

Intracellular ATP molecules generated during carbohydrate metabolism increased insulin secretion in a K_ATP_ channel-dependent manner. It was previously reported that extracellular ATP (200 μM) can increase insulin secretion in pancreatic cells^33, 34^. However, whether extracellular ATP increases insulin in a K_ATP_ channel-dependent manner remains unknown. The loss of function of SUR1 protein in *ABCC8*-deficient cells was correlated with inactivation of K_ATP_ channels. Therefore, we tested the fold change of C-peptide secretion after treatment with extracellular ATP. We observed a 1.6-fold increase in C-peptide secretion in all three types of cells (Fig. 1H). As extracellular calcium increases insulin secretion^35^, we next sought to determine whether there is any impact of *ABCC8* mutation on calcium chloride (10 mM)-mediated insulin secretion. We found a positive role of calcium chloride on insulin secretion with an approximately 2.9-fold increase in the three types of cells (Fig. 1H). To further elucidate the mechanism of insulin secretion, the role of sodium-potassium adenosine triphosphatase or the Na-K pump was investigated. The Na-K pump is located in the plasma membrane of all animal cells and functions to pump sodium outward and potassium inward. Ouabain increases insulin secretion as an Na-K pump inhibitor^36, 37^. However, it remains unknown whether the insulin secretion increased by ouabain is dependent on K_ATP_ channels. Our findings indicated an overall of 1.4-fold increase in insulin secretion by wild-type and *ABCC8*-deficient cells treated with ouabain (Fig. 1H). In conclusion, our results demonstrated that extracellular ATP, calcium and ouabain increase insulin secretion in a K_ATP_ channel-independent manner.

### Insulin secretion rate of *ABCC8*-deficient cells is more sensitive to extracellular potassium

K_ATP_ channels play an important role in the regulation of insulin secretion and the maintenance of intracellular potassium homeostasis. To investigate the relation of SUR1 with potassium, we measured insulin secretion by *ABCC8-*deficient and wild-type cells in different concentrations of extracellular potassium: low potassium, normal potassium and high potassium environments.

KRBH buffer was derived from Ringer’s solution^38^ to mimic body fluids and was thus selected as the medium in which to measure insulin secretion. We defined KRBH buffer as the normal potassium medium with a K^+^ concentration of 5.9 mM (4.7 mM KCl and 1.2 mM KH_2_PO_4_). KRBH buffer supplemented with 30 mM KCl^19^ is widely used to induce insulin secretion by differentiated insulin-producing cells and was defined as the high potassium medium with a K^+^ concentration of 35.9 mM (34.7 mM KCl and 1.2 mM KH_2_PO_4_). KRBH buffer without KCl was used as the low potassium medium with a K^+^ concentration of 1.2 mM (1.2 mM KH_2_PO_4_).

**Figure 2:**
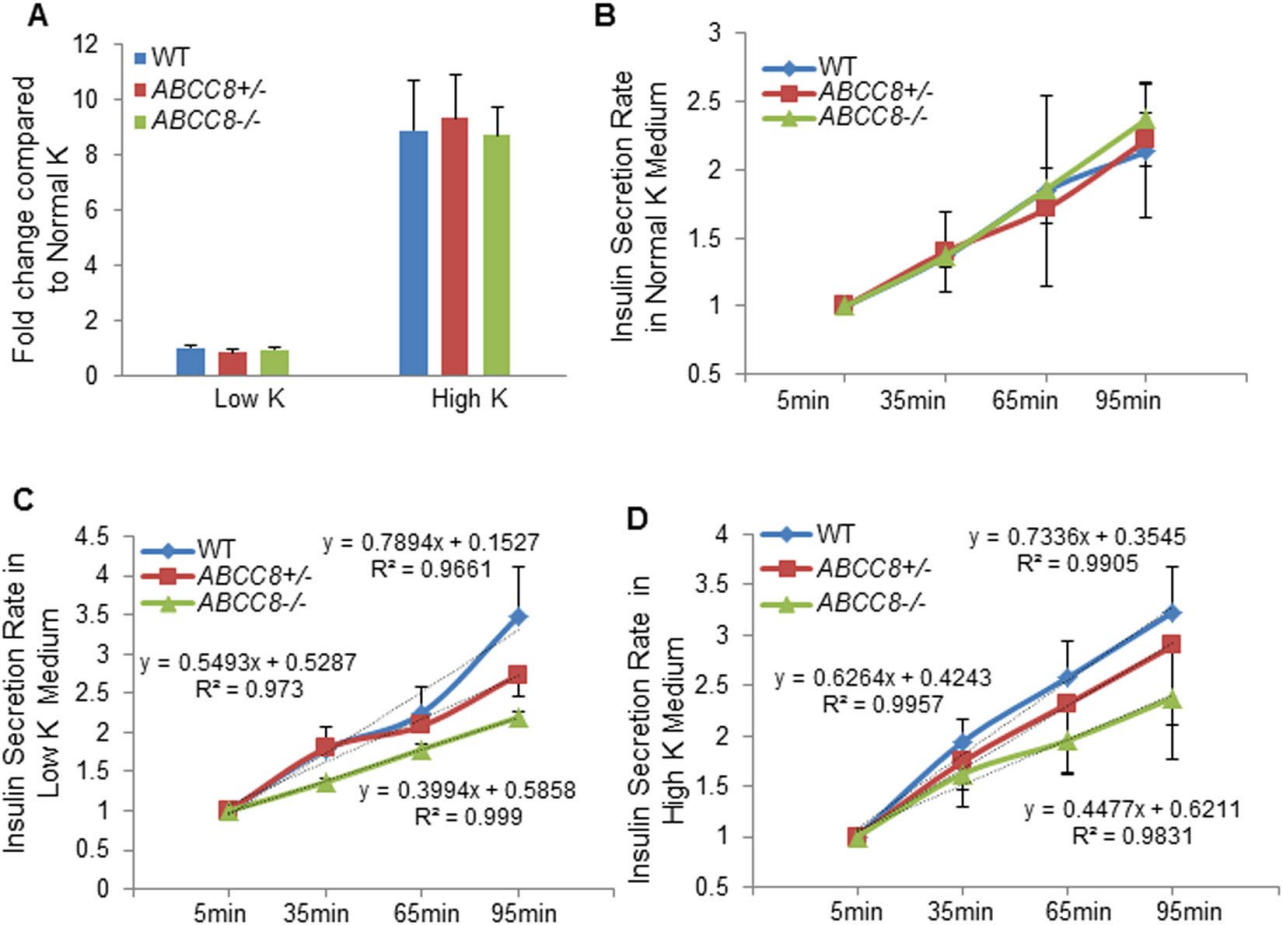
*ABCC8* mutation decreased the insulin secretion rate in low and high potassium medium. (**A**) The fold change of C-peptide content after incubation in low K^+^ and high K^+^ medium compared with normal K^+^ medium. Wild-type, heterozygous mutated and homozygous mutated cells exhibited similar changes. (**B**) Insulin secretion rate in normal K^+^ medium (KRBH). Wild-type and *ABCC8*-deficient cells had similar secretion rates. (**C**) Insulin secretion rate in low K^+^ medium. *ABCC8* mutation decreased the insulin secretion rate in low K^+^ medium. (**D**) Insulin secretion rate in high K^+^ medium. *ABCC8* mutation decreased the insulin secretion rate in high K^+^ medium.

We first measured insulin secretion in different potassium media for a pre-defined time period (35 min) and calculated the fold change of low K^+^/normal K^+^ and high K^+^/normal K^+^. We observed that, compared with normal potassium medium (KRBH), low potassium medium had no distinct effect, corresponding to an approximately 1-fold change in insulin secretion for all cells. On the other hand, an increase in insulin secretion by 8.8-fold was observed for all cells in the high potassium medium (Fig. 2A).

Finally, dynamic secretion curves in different potassium environments within 95 min were plotted by measuring insulin levels at 30-min intervals. In the normal potassium medium, wild-type and *ABCC8*-deficient cells demonstrated similar insulin secretion rates (Fig. 2B), whereas the insulin secretion rate of *ABCC8* ^−/−^ cells decreased in low (slope: 0.3994 vs 0.7894) (Fig. 2C) and high potassium media (slope: 0.4477 vs 0.7336) (Fig. 2D) compared with wild-type cells. The slopes of the curves for *ABCC8* ^+/−^ cells in the low and high media were between those for the wild-type and *ABCC8* ^−/−^ cells, corresponding to 0.5493 and 0.6264, respectively. These results indicated that the *ABCC8* mutation causes a decreased insulin secretion rate in low and high potassium environments (Fig. 2C,D).

## Discussion

In this report, we recapitulated the clinical phenomenon of CHI. *ABCC8-*deficient stem cells-derived insulin-producing cells exhibited higher insulin secretion than their isogenic wild-type cells. More importantly, excess insulin secretion can be rescued by drugs used to treat clinical CHI. Our results demonstrated the ability to model the phenotype of excess insulin secretion of CHI stem cells *in vitro*. The structure of K_ATP_ channels^39, 40^ was recently reported, providing new targets for drug design. Our CHI stem cell model represents a highly promising choice for testing such candidate drugs *in vitro*.

Although many attempts have been made to generate insulin-producing cells from human pluripotent stem cells in the past decade, insulin-producing cells resemble fetal rather than adult β cells^41^, which lack or only exhibit partial function of glucose-stimulated insulin secretion compared with primary β cells. Insulin secretion is tightly linked with glucose metabolism. In K_ATP_ CHI, the precise regulation of blood glucose and insulin secretion is lost due to the malfunction of K_ATP_ function. Therefore, immature β cells differentiated from stem cells don’t play a key role in CHI research.

Diazoxide has been proven to be effective in the treatment of hypoglycemia in some CHI patients. However, not all patients are responsive to diazoxide. In our model system, the insulin-producing cells lost the ability to respond to diazoxide when SUR1 function was completely lost (*ABCC8* ^−/−^). This cell line could be utilized to screen for drugs to treat diazoxide-unresponsive CHI patients.

Insulin is important in maintaining potassium homeostasis which plays critical role in many physiological processes. Insulin prevents substantial changes in extracellular fluid (ECF) K^+^ concentration as it promotes potassium to transfer from ECF to intracellular fluid (ICF) by increasing Na^+^/K^+^-ATPase activity independent of glucose uptake^42^. Increases in plasma potassium concentrations stimulate pancreatic insulin secretion^43^, which promotes a shift of excess K^+^ into the intracellular compartment. Direct depolarization by the addition of potassium chloride (hyperkalemia) consistently increases insulin secretion by insulin-producing cells differentiated from pluripotent stem cells^19, 44, 45^. K_ATP_ channels favor depolarization and then enhance calcium-mediated insulin secretion. K_ATP_ channels consist of two components, SUR1 and Kir6.2. In this report, we demonstrated that *ABCC8*-deficient insulin-producing cells exhibit a decreased insulin accumulation rate in high potassium medium. Zeng has reported that Kir6.2 deficiency impaired insulin secretion in response to high concentrations of potassium chloride^46^, indicating that SUR1 and Kir6.2 play different roles in response to high potassium concentrations. The role of low potassium (hypokalemia) in insulin secretion remains controversial. Some studies have shown that hypokalemia could reduce insulin secretion^47, 48^, but others have shown that clamping plasma potassium levels potentiates the insulin secretory response to glucose^49^. However, the dynamic secretion curve has not yet been reported. Our results showed that malfunction of SUR1 decreased the insulin secretion rate.

Although we successfully constructed a CHI stem cell model, many questions remain to be answered. CHI is a complicated disorder with unregulated insulin secretion. However, there are still approximately 50% patients whose genetic abnormalities have not been elucidated^4^. Induced pluripotent stem (iPS) cells^50, 51^ generated from somatic cells with several transcript factors provide the opportunity to produce various disease-specific cell types^52^ and, therefore, are an attractive method for modeling CHI. One possibility is collecting urine cells from these patients and reprograming them into iPS cells^53^ to generate a “disease-in-a-dish” model and then continuing with specific differentiation. Understanding of these innovative mechanisms will provide profound knowledge regarding the function of the pancreatic β-cells and novel treatments for CHI and even for diabetic mellitus.

In conclusion, we captured the CHI phenotype of excess insulin secretion with *ABCC8*-deficient ES cells that were established in our lab. We further investigated the role of SUR1 in insulin secretion in different potassium media. Our study not only provides an attractive model for *in vitro* CHI research but may provide a platform for studying other related hereditary pancreatic diseases.

## Methods

### Ethical statement

The cell lines used in this report were approved by the Ethics Committee of Guangzhou Institutes of Biomedicine and Health, Chinese Academy of Sciences.

### Cell culture

Human embryonic H1 stem cells and *ABCC8-*deficient stem cells^17, 18^ free of mycoplasma were routinely maintained on Matrigel (Becton Dickinson) with mTeSR1 (Stemcell). The cells were passaged for 4 days with accutase (Sigma). Rock inhibitor (10 μM; Y27632, Selleck) was added to improve the survival rate to 24 h after replating.

### Pancreatic differentiation

For pancreatic differentiation, we followed Yuya Kunisada’s protocol with slight modification^19^. Briefly stem cells were passaged on Matrigel-coated 24-well plates at a density of 10 × 10^4^ cells per well. Stem cells were cultured with mTeSR1 including Y27632 for 24 h and then 3 days with mTeSR1 to nearly reach confluence. For differentiation, the cells were cultured in RPMI1640 (Gibco) containing B27 insulin (Gibco), 100 ng/mL activin A (Pepro Tech) and 3 μM CHIR99021 (Stemgent) for 24 h and for 48 h in RPMI 1640 containing B27 minus insulin and 100 ng/mL activin A. Subsequently, the media was changed with DMEM/F12 containing B27 (Gibco), 1 μM dorsomorphin (Calbiochem), 10 μM SB431542 (Sigma) and 2 μM retinoic acid (Sigma) for 7 days and medium was replaced every other day. For insulin-producing cell differentiation, the medium was changed to DMEM/F12 containing B27, 10 μM forskolin (Stemgent), 10 μM dexamethasone (Enzo Life Sciences), 5 μM Alk5 inhibitor II (Calbiochem) and 10 mM nicotinamide (Stemcell) for 12 days and provided with fresh medium every other day.

### Immunofluorescence microscopy

The cells were fixed in 1% paraformaldehyde for 30 min. After washing 3 times with PBS, the cells were blocked and permeabilized in blocking solution (PBS containing 3% bovine albumin and 0.2% Triton X-100) for 30 min at room temperature. The cells were then incubated with primary antibodies in blocking solution at 4 °C overnight, washed 3 times, and incubated with the corresponding secondary antibodies for 1 h at room temperature. The cells were washed twice and stained with DAPI (Sigma) for 5 min and then analyzed using a Leica DMI6000B microscope (Leica Microsystems). Primary antibodies used in this study were as follows: FoxA2 (R&D, AF2400, 1:200), Sox17 (RD, AF1924, 1:200), goat anti-Pdx1 (R&D, AF2419, 1:200), guinea pig anti-insulin (Dako, A056401, 1:300), and rabbit anti-C-peptide (Abcam, ab14181, 1:300).

### Flow cytometry

Single-cell suspensions of differentiated human ES cell cultures were obtained by dissociating cells with 0.25% trypsin, fixing with 2% paraformaldehyde and permeabilizing with BD Perm/Wash buffer (Becton Dickinson). The cells were incubated with guinea pig anti-insulin antibody (1:1000, Dako) for 30 min at room temperature and then stained with Alexa Fluor 488-conjugated goat antibody directed against guinea pig (1:800) for 30 min at room temperature. Flow cytometry was performed using an Accuri C6 system (Becton Dickinson).

### Insulin/C-peptide release assay by ELISA

To measure insulin secretion, differentiated cells were pre-incubated on day 22 for 1 h at 37 °C in Krebs-Ringer bicarbonate HEPES buffer (KRBH buffer,116 mM NaCl, 4.7 mM KCl, 2.5 mM CaCl_2_ 1.2 mM KH_2_PO4, 1.2 mM MgSO_4_, 24 mM HEPES, 25 mM NaHCO_3_, and 0.1% BSA). Cells were incubated at 37 °C in KRBH for 1 h, and the supernatant was collected. Next, insulin and C-peptide were measured using human insulin ELISA Kit (Millipore) and a C-peptide ELISA kit (Millipore), respectively, according to the manufacturer’s instructions. To determine total protein content, cells were lysed with RIPA lysis buffer, and protein content was measured with a BCA Protein Assay Kit (Millipore). The amount of insulin or C-peptide was normalized to the amount of total protein in the corresponding cell lysate.

To measure the effect of diazoxide and glimepiride on insulin secretion, differentiated cells were pre-incubated with KRBH buffer at 37 °C for 1 h on day 22 and then incubated with KRBH buffer containing DMSO for 1 h at 37 °C; the supernatant was collected. Then, the same cells were incubated with KRBH buffer containing 100 μM diazoxide (Tocris) or 10 μM glimepiride (Selleck), and the supernatant was collected. Both diazoxide and glimepiride were dissolved in DMSO. The C-peptide levels in both supernatants were measured with human C-peptide ELISA kit (Millipore). To measure the content of total protein, cells were lysed with RIPA lysis buffer, and the total protein content was determined with a BCA Protein Assay Kit (Millipore). The amount of insulin or C-peptide was normalized to the amount of total protein in the corresponding cell lysate. The ratios of C-peptide content for cells treated with diazoxide/DMSO and glimepiride/DMSO were measured as fold changes.

To test the rescue effect of octreotide, nicorandil and nifedipine on insulin secretion, after pre-incubation with KRBH buffer at 37 °C for 1 h, differentiated cells on day 22 were incubated with KRBH buffer containing vehicle for 1 h at 37 °C, and the supernatant was collected. Then, the same cells were incubated with KRBH buffer containing 50 μg/mL octreotide acetate (Selleck, dissolved in H_2_O), 200 μM nicorandil (Selleck, dissolved in DMSO), or 50 μM nifedipine (Selleck) for 1 h at 37 °C, and the supernatant was collected. The ratios of C-peptide content for cells treated with octreotide/H_2_O, nicorandil/DMSO or nifedipine/DMSO were measured as fold changes.

To test the effect of extracellular ATP, calcium ions and ouabain on insulin secretion, after pre-incubation with KRBH buffer at 37 °C for 1 h, differentiated cells on day 22 were incubated with KRBH buffer containing vehicle, and the supernatant was collected. Then, the same cells were incubated with KRBH buffer containing 200 μM ATP (Selleck) 10 mM calcium chloride (CaCl_2_, Aladdin) or 100 μM ouabain (Selleck) for 1 h at 37 °C, and the supernatant was collected. The ratios of C-peptide content for cells treated with ATP/H_2_O, calcium chloride/H_2_O or ouabain/DMSO were measured as fold changes.

To measure the insulin secretion response to different potassium environments, differentiated cells on day 22 were incubated for 35 min at 37 °C in KRBH buffer (normal potassium medium, K^+^ concentration of 5.9 mM) after pre-incubation with KRBH buffer for 1 h. The cells were then incubated in low potassium medium (KRBH buffer without KCl, K^+^ concentration of 1.2 mM) and high potassium medium (KRBH buffer plus 30 mM KCl, K^+^ concentration of 35.9 mM). The ratios of C-peptide content for incubation in low potassium/normal potassium and high potassium/normal potassium were measured as fold changes.

To measure the insulin secretion rate in low, normal and high potassium environments, on day 22, differentiated cells were pre-incubated with KRBH buffer for 1 h and then individually incubated in 3 types of medium at 37 °C. The supernatant was collected at 5 min, 35 min, 65 min and 95 min. The ratios of C-peptide content corresponding to 5 min/5 min, 35 min/5 min, 65 min/5 min, and 95 min/5 min were reported as fold changes. A linear secretion curve was plotted according to the fold change. The fitted line was valid when the R^2^ value was greater than 0.95. The slope of the straight line was measured as the insulin secretion rate.

### Statistical analysis

Immunofluorescence microscopy and flow cytometry analyses were performed three times, and a representative group was selected. All ELISA experiments were independently performed 3 times. Data are shown as the mean ± SD. Statistical differences between the three groups were evaluated using one-way ANOVA and the Bonferroni *post hoc* test. Differences were considered significant when the P value was less than 0.05 (*) and highly significant when the P value was less than 0.01 (**).

### Data availability statement

All data generated or analysed during this study are included in this published article (and its Supplementary Information files).

## Electronic supplementary material

Supplementary Figure 1
