# Supplementary material for: Modeling Congenital Hyperinsulinism with ABCC8-Deficient Human Embryonic Stem Cells Generated by CRISPR/Cas9
Source: Sci Rep. 2017 Jun 9;7:3156. doi: 10.1038/s41598-017-03349-w (PMC5466656; doi:10.1038/s41598-017-03349-w)
Supplement: Supplementary file 1 — Supplementary Figure 1 [file 41598_2017_3349_MOESM1_ESM.pdf]

Modeling Congenital Hyperinsulinism with *ABCC8* Deficient Human Embryonic Stem Cells Generated by CRISPR/Cas9

Dongsheng Guo[1,2,3,4], Haikun Liu[1,3,4], Aynisahan Ruzi[1,2,3,4], Ge Gao[1,3,4], Abbas Nasir[1,2,3,4], Yanli Liu[1,2,3,4], Fan Yang[1,3,4], Feima Wu[1,2,3,4], Guosheng Xu[1,2,3,4], Yin-xiong Li[1,2,3,4 *]

[1]Institute of Public Health, Guangzhou Institutes of Biomedicine and Health, Chinese Academy of Sciences, Guangzhou, China;

[2]University of Chinese Academy of Sciences;

[3]Key Laboratory of Regenerative Biology, South China Institute for Stem Cell Biology and Regenerative Medicine, Guangzhou Institutes of Biomedicine and Health, Chinese Academy of Sciences, Guangzhou, China;

[4]Guangdong Provincial Key Laboratory of Biocomputing, Guangzhou Institutes of Biomedicine and Health, Chinese Academy of Sciences, Guangzhou, China;

Contact information for correspondence: Yin-xiong Li, Guangzhou Institutes of Biomedicine and Health, Chinese Academy of Sciences, 190 Kaiyuan Avenue, Science Park, Guangzhou, Guangdong, 510530, China;

Email: li_yinxiong@gibh.ac.cn;

Supplementary Figure 1

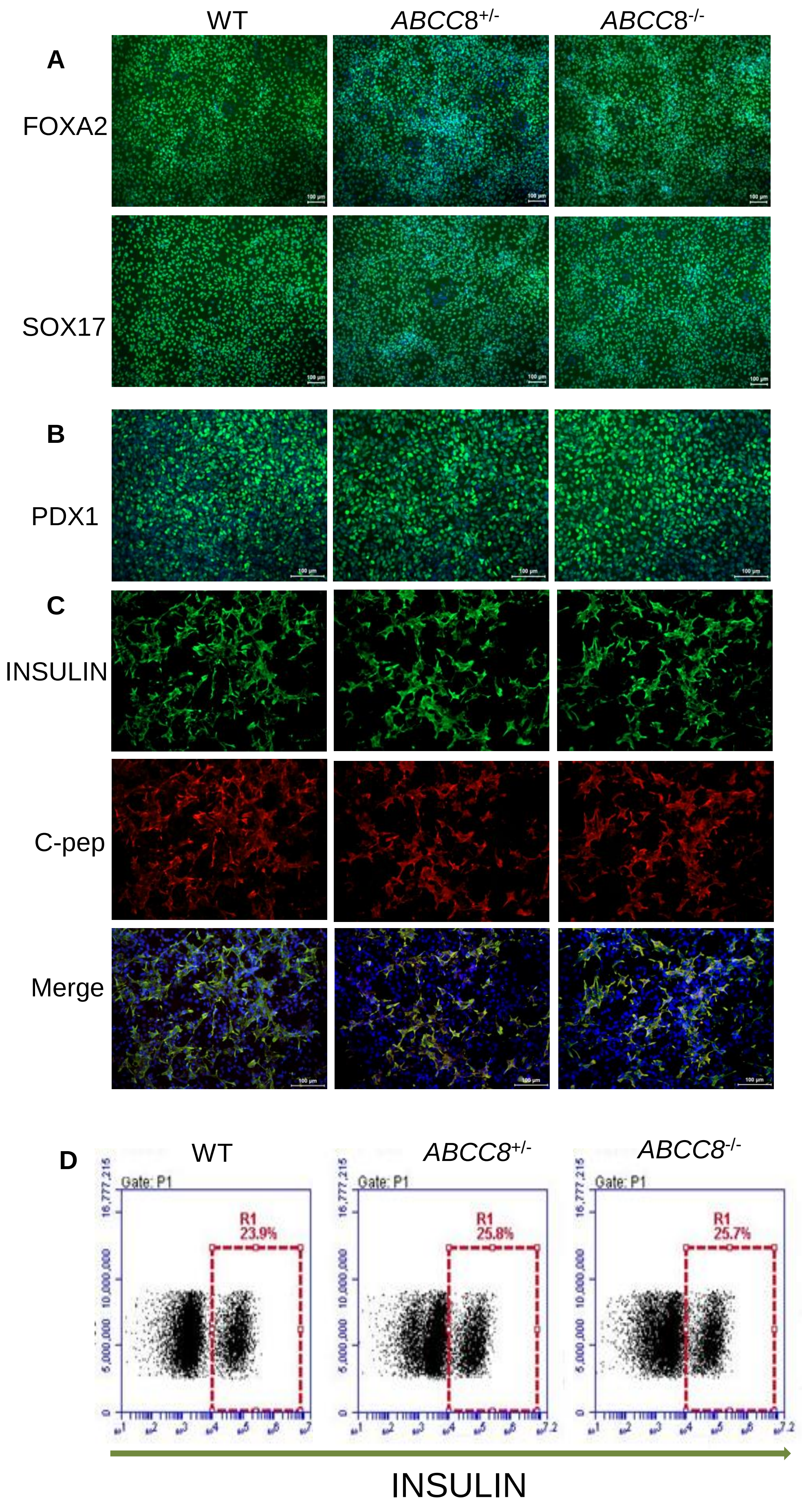

Supplementary Figure 1 Differentiation of stem cells into insulin producing cells

A. Immunofluorescence for FOXA2 and SOX17 at definitive endoderm (DE) stage of differentiation.

B. Immunofluorescence for PDX1 at pancreatic progenitor stage of differentiation.

C. Immunofluorescence for Insulin and C-peptide at insulin producing cells stage of differentiation.

D. FACS analysis for insulin at insulin producing cells stage of differentiation.
